# Evaluation of the Effects of *Astragalus membranaceus* on the Pharmacokinetics of Pemetrexed Disodium and Gemcitabine in Rats by a Simple High-Performance Liquid Chromatography/UV Method

**DOI:** 10.1155/2019/3162426

**Published:** 2019-04-28

**Authors:** Zixuan Chu, Zhiyuan Wang, Teng Liu, Shan Xiong, Bin Liu

**Affiliations:** ^1^School of Medicine and Life Sciences, University of Jinan-Shandong Academy of Medical Sciences, Jinan, Shandong 250200, China; ^2^Institute of Materia Medica, Shandong Academy of Medical Sciences, Jinan, Shandong 250062, China; ^3^Key Laboratory for Biotech-Drugs Ministry of Health, Jinan, Shandong 250062, China; ^4^Key Laboratory for Rare & Uncommon Diseases of Shandong Province, Jinan, Shandong 250062, China; ^5^The People's Hospital of Yucheng, Yucheng, Shandong 251200, China

## Abstract

Combination therapy is opted as a potential therapeutic strategy for cancer treatment. *Astragalus membranaceus* combined with pemetrexed disodium or gemcitabine could reinforce the overall effects and alleviate the adverse effects. To investigate the effects of *Astragalus membranaceus* on the pharmacokinetics of pemetrexed disodium and gemcitabine, a HPLC method for simultaneous determination of pemetrexed disodium and gemcitabine in rat plasma was developed and validated. Chromatographic separation was achieved on a C18 column using a gradient mode containing water (containing 20 mM NaH_2_PO_4_ and 0.1% FA) and methanol at a flow rate of 0.8 mL/min. The specificity, linearity, recovery, stability, precision, and accuracy of the HPLC method were all validated. The rats were pretreated with *Astragalus* extract at the dosage of 3 g/kg for 20 consecutive days until we commence studying the pharmacokinetics of pemetrexed disodium or gemcitabine. There were no significant differences in pharmacokinetic parameters of pemetrexed disodium between the *Astragalus* extract treatment group and the control group. However, AUC, MRT, and Cl of gemcitabine were changed dramatically after treating with *Astragalus* extract (*p* < 0.05). The AUC_(0−*t*)_, AUC_(0−*∞*)_, and MRT of gemcitabine decreased from 15747.12 ± 497.11 to 12312.41 ± 594.21 mg/L·min, 15976.18 ± 511.33 to 12489.59 ± 682.01 mg/L·min, and 97.83 ± 5.82 to 84.37 ± 2.79 min, respectively. The Cl of gemcitabine increased from 0.019 ± 0.0067 to 0.024 ± 0.0013 L/min/kg. The results showed that the pretreatment of *Astragalus* extract could exert an influence on the pharmacokinetic characteristics of gemcitabine in rats.

## 1. Introduction

For the past few years, the incidence of malignant tumor has been increasing rapidly with the changes of lifestyles. Surgery, radiotherapy, and chemotherapy are the most common and effective ways to the treatment of malignant tumors. Pemetrexed and gemcitabine are both highly effective anticancer drugs [1]. Pemetrexed disodium is a multitargeted antifolate, which inhibits various folate-dependent enzymes, including thymidylate synthase, dihydrofolate reductase, and glycinamide ribonucleotide formyltransferase [2]. Gemcitabine, a specific analog of deoxycytidine, inhibits ribonucleotide reductase [3].

However, there are many adverse reactions of the two drugs such as nephrotoxicity, myelosuppression, and gastrointestinal reaction. These adverse reactions limit their clinical application, and furthermore, the descent of immunity caused by pemetrexed and gemcitabine reduces the efficacy. Consequently, it is imperative to find a way to minimize the occurrence of adverse reactions and ensure the efficacy [4, 5].

Combination therapy in cancer is now opted as a potential therapeutic strategy for cancer treatment [6]. In the recent years, traditional Chinese medicine (TCM) has made great progress in the clinical application and experimental study of adverse reactions to radiotherapy and chemotherapy [7]. For instance, *Astragalus membranaceus* plays a vitally important role in the prevention and treatment of cancer [8]. *Astragalus membranaceus*, known as Huangqi in China, is the dried root of *Astragalus membranaceus* (Fisch.) Bge. or *Astragalus membranaceus* (Fisch.) Bge. var. *mongholicus* (Bge.) Hsiao and has been shown to contain triterpene saponins, isoflavonoids, polysaccharides, amino acids, and some trace elements [9]. It has been utilized to reinforce Qi, to strengthen the superficial resistance, and to promote the discharge of pus and the growth of new tissue [10]. In the study of modern medicine, twenty active ingredients of drugs were screened out from *Astragalus membranaceus*. Fourteen targets have been identified to be associated with cancer, which played an therapeutic role in treating cancer by regulating target proteins, such as erb-b2 receptor tyrosine kinase 2 (ERBB2), androgen receptor (AR), SRC proto-oncogene, nonreceptor tyrosine kinase (SRC), and epidermal growth factor receptor (EGFR), estrogen receptor 1 (ESR1), as well as proteoglycans in cancer, cancer pathways, and microRNAs in cancer and other pathways [11]. Additionally, it was reported that *Astragalus membranaceus* combined with pemetrexed disodium or gemcitabine had synergistic effects, which reinforced the overall effects and alleviated the adverse effects [12].

The aim of this study was to investigate whether the co-administration of *Astragalus* extract would alter the pharmacokinetic characteristics of pemtrexed disodium or gemcitabine. Based on what is mentioned above, a highly sensitive, specific, simple, and accurate method for the simultaneous quantitation of pemetrexed disodium and gemcitabine in rat plasma by HPLC was established and validated.

## 2. Materials and Methods

### 2.1. Reagents and Chemicals

**Figure 1:**
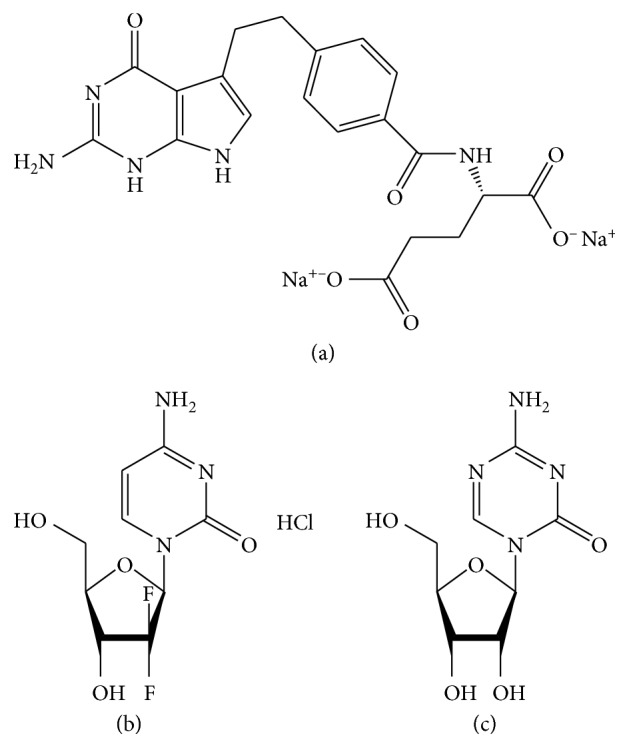
Chemical structures of pemetrexed disodium (a), gemcitabine hydrochloride (b), and IS (c).

Pemetrexed disodium and gemcitabine hydrochloride (Figures 1(a) and 1(b)) were supplied by the National Institute for the Control of Pharmaceutical and Biological Products (Beijing, China). Azacitidine (internal standard, IS, Figure 1(c)) was obtained from Dalian Meilun Biotech Co., Ltd. *Astragalus* extract (10 : 1 aqueous extract) was purchased from Shaanxi Sciphar Natural Products Co., Ltd. HPLC-grade methanol and HPLC-grade acetonitrile were obtained from Tedia (Fairfield, OH, USA). All other chemicals were of analytic grade or better.

### 2.2. Animals

All animal studies were approved by the institutional ethics committee prior to the study. Twenty male Sprague-Dawley (SD) rats weighing 250 ± 20 g were obtained from Shandong Laboratory Animal Center of Shandong Academy of Medical Sciences (Certificate no. SCXK (Shandong) 2014-0007). Rats were housed in cages with 12 h day light/12 h night cycle, a temperature of 25 ± 2°C, and relative humidity of 65 ± 5%. All rats were fed with standard rat diet and had free access to water.

### 2.3. Equipment and HPLC Conditions

The chromatographic analysis was carried out on an UltiMate 3000 ultraperformance liquid chromatography system (Thermo Scientific, USA). Chromatographic separation was carried out at 30°C on a Thermo Hypersil GOLD (Thermo Fisher Scientific, Waltham, MA, USA) C18 column (150 mm × 4.6 mm, 5 *μ*m). The mobile phase consisted of water (containing 20 mM NaH_2_PO_4_ and 0.1% FA) and methanol at a flow rate of 0.8 mL/min. The initial methanol content was 2.5%. Elution was in a linear gradient, with methanol content changing from 2.5% to 95% between 4.0 and 6.0 min. Methanol content was decreased to 2.5% within 2.1 min. The total run time was 13.0 min. The detection wavelengths were 250 nm and 268 nm for pemetrexed disodium and gemcitabine, respectively. All the operations, the acquiring and analysis of data, were controlled using Chromeleon software, version 7.2.2.6686 (Thermo Scientific, USA).

### 2.4. Stock Solutions, Calibration Standards, and Quality Control Samples

The stock standard solutions of pemetrexed disodium and gemcitabine were both prepared with dimethyl sulfoxide (DMSO) at the concentrations of 10.0 mg/mL and then mixed and serially diluted with methanol to obtain the working solutions. The IS solution of 4 mg/mL was prepared in DMSO. All standard solutions were kept at 4°C before use.

The diluted solutions were prepared by spiking appropriate amounts of the standard solutions in blank rat plasma at final concentrations of 0.5, 5, 20, 100, 250, and 500 *μ*g/mL for both pemetrexed disodium and gemcitabine. The calibration equations were calculated by the least-squares linear regression method.

The quality control (QC) samples were prepared in blank plasma at four different concentration levels, lower limit of quantification (LLOQ, 0.5 *μ*g/mL), low QC (LQC, 1 *μ*g/mL), medium QC (MQC, 200 *μ*g/mL), and high QC (HQC, 400 *μ*g/mL), for both pemetrexed disodium and gemcitabine.

### 2.5. Plasma Sample Preparation

After thawing the plasma samples at room temperature, the standard protein precipitation method was applied to prepare the samples. 50 *μ*L plasma was mixed with 10 *μ*L IS and 50 *μ*L 10% trichloroacetic acid. The tubes were vortex-mixed for 60 s and then centrifuged at 14 000 rpm for 10 min at 4°C, and 20 *μ*L supernatant was then injected into the HPLC system for analysis.

### 2.6. Method Validation

Validation procedures were carried out according to the China Food and Drug Administration guidelines for preclinical pharmacokinetic study with respect to selectivity, linearity, precision, accuracy, recovery, dilution integrity, and stability [13].

### 2.7. Pharmacokinetic Experiment

Twenty SD rats were randomly assigned to four groups (*n*=5). The rats in group I and group III were given *Astragalus* extract at the dosage of 3 g/kg for 20 consecutive days by gavage; meanwhile, the rats in the other two groups were given corresponding volume of purified water under the same conditions. On day 21, all rats were fasted overnight with free access to water for at least 12 h. The rats in groups I and II were administered an intravenous dose (300 mg/kg *via* the tail vein) of pemetrexed disodium. The rats in groups III and IV were given gemcitabine at a single dosage of 150 mg/kg by the same way [1, 2]. About 150 *μ*L of blood sample was collected in a centrifuge tube before and at 2, 5, 15, 30, 60, 120, 240, 360, and 480 min after administration, and the heparin was used as an anticoagulant. All the blood samples were centrifuged at 3000 rpm for 15 min at 4°C, and the plasma samples were stored at −20°C for later determination of pemetrexed disodium and gemcitabine levels [14, 15].

### 2.8. Statistics

Statistical analyses were performed with SPSS 20.0. Difference of means between two groups were analysed using the Student's *t*-test. A value of *p* < 0.05 was considered as statistically significant.

## 3. Results and Discussion

### 3.1. Method Development

An appropriate IS is beneficial to improve the quantitative accuracy. Azacitidine was selected as the IS, which has the similar structure to gemcitabine and did not interfere with pemetrexed disodium or gemcitabine at the retention times with stable response. The mobile phase consisted of water (containing 20 mM NaH_2_PO_4_ and 0.1% FA) and methanol with gradient elution, and Thermo Hypersil GOLD C18 (150 mm × 4.6 mm, 5 *μ*m) column played a great role in achieving good peak symmetry with appropriate retention time and separation.

### 3.2. Method Validation

#### 3.2.1. Selectivity

**Figure 2:**
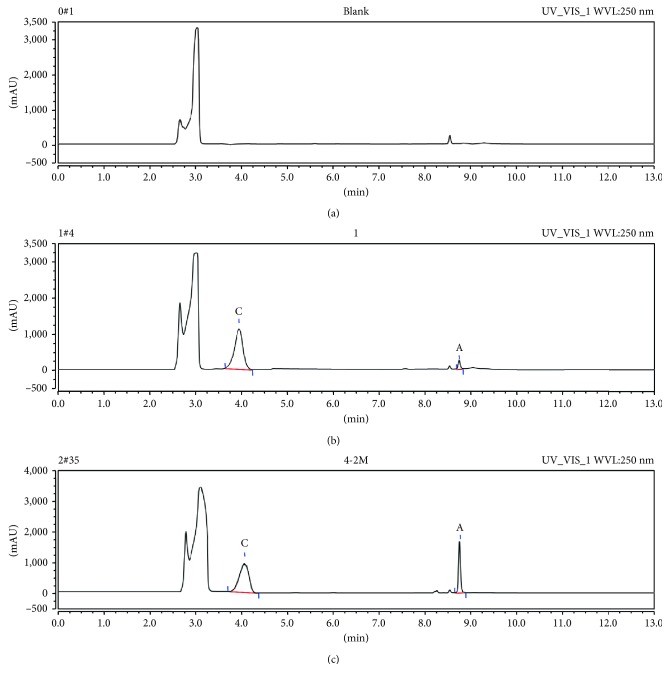
Representative chromatograms of pemetrexed disodium (A) and IS (C) in rat plasma at 250 nm. Chromatographic profile of (a) a blank plasma sample, (b) a sample of plasma spiked with analytes and IS, and (c) a sample of plasma at 2 min after intravenous administration of pemetrexed disodium.

**Figure 3:**
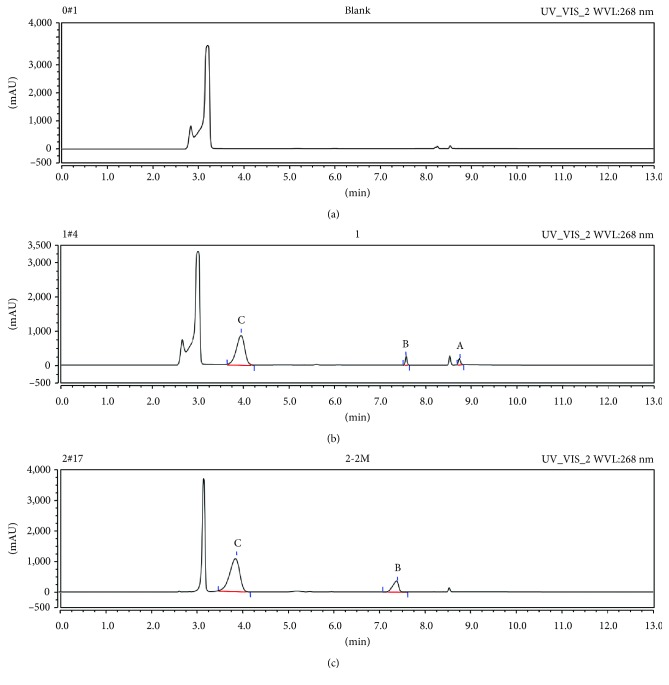
Representative chromatograms of pemetrexed disodium (A), gemcitabine hydrochloride (B) and IS (C) in rat plasma at 268 nm. Chromatographic profile of (a) a blank plasma sample, (b) a sample of plasma spiked with analytes and IS, and (c) a sample of plasma at 2 min after intravenous administration of gemcitabine.

The selectivity was tested by comparing the chromatograms of the blank plasma sample, blank plasma spiked with pemetrexed disodium, gemcitabine, and IS and plasma after intravenous injection of pemetrexed disodium or gemcitabine at 250 nm and 268 nm. No interferences from endogenous substances were observed around the retention times of pemetrexed disodium, gemcitabine, or IS. The retention times of pemetrexed disodium, gemcitabine, and IS were 8.747, 7.577, and 3.953 min, respectively (Figures 2 and 3).

#### 3.2.2. Linearity and LLOQ

The calibration of pemetrexed disodium and gemcitabine showed linear relationship over the concentration range of 0.5–500 *μ*g/mL. The regression equations in rat plasma were *Y* = 0.1113*X* − 2.1728 (*r*^2^ = 0.9971) and *Y* = 0.1782*X* + 0.0502 (*r*^2^ = 0.9956) for pemetrexed disodium and gemcitabine, respectively, where *Y* meant the peak area ratio of analytes to IS and *X* equalled the nominal concentration of analytes. In the present study, the precision (RSD) of LLOQ (0.5 *μ*g/mL) was within ±20% from the theoretical value.

#### 3.2.3. Precision and Accuracy

For precision and accuracy of the method, QC samples at three concentration levels (LQC, MQC, and HQC) were assayed in five replicates on three consecutive days. Values within ±15% for precision and accuracy were considered acceptable. The results of precision and accuracy measurements are presented in Table 1, which were completely within the acceptance limits.

#### 3.2.4. Recovery and Dilution Integrity

The recovery was assessed by comparing the responses from QC samples at three concentration levels with the responses of pemetrexed disodium and gemcitabine from neat samples at the same concentrations. The recovery was high, consistent, and reproducible with the values from 88.16% to 103.15% for pemetrexed disodium and from 93.44% to 99.99% for gemcitabine, respectively.

Six replicate samples, with the concentrations of 1000 and 1000 *μ*g/mL for pemetrexed disodium and gemcitabine, respectively, were diluted 5-fold with blank rat plasma. The diluted samples were analysed and estimated accuracy and precision compared to the theoretical value. The precision (RSD) was less than 15%, and the accuracy was within 85–115% for all the analytes.

#### 3.2.5. Stability

The stability of pemetrexed disodium and gemcitabine was investigated by analyzing QC plasma samples at LQC and HQC under different conditions. The temperature and timing conditions were as follows: 6 h at room temperature, stored at −20°C for 7 d, and 24 h at 4°C in storehouse. The results indicated that analytes were stable under conditions investigated in the study (Table 2).

### 3.3. Pharmacokinetic Experiment

**Figure 4:**
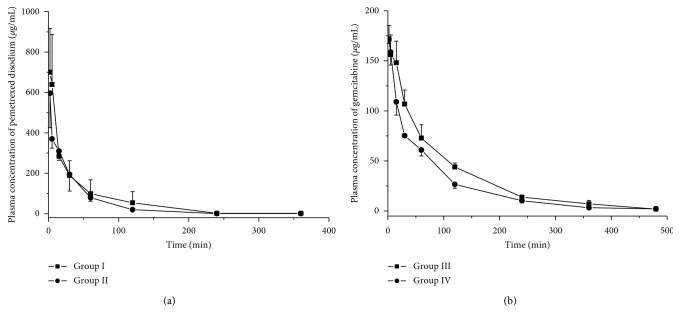
Plasma concentration-time profiles of pemetrexed disodium (a) and gemcitabine (b) following single intravenous administration in rats (*n*=5). Group I: rats were intravenously administered with 300 mg/kg pemetrexed disodium. Group II: rats were intravenously administered with 300 mg/kg pemetrexed disodium at day 21 of treatment with 3 g/kg/day *Astragalus* extract. Group III: rats were intravenously administered with 150 mg/kg gemcitabine. Group IV: rats were intravenously administered with 150 mg/kg gemcitabine at day 21 of treatment with 3 g/kg/day *Astragalus* extract.

The plasma concentration-time profiles of pemetrexed disodium and gemcitabine following single intravenous administration in rats are presented in Figure 4. The main pharmacokinetic parameters of pemetrexed disodium and gemcitabine were processed by noncompartmental model using the DAS 2.0 software package (Mathematical Pharmacology Professional Committee of China, Shanghai, China). The pharmacokinetic parameters including AUC_(0−*t*)_, AUC_(0−*∞*)_, MRT, t_1/2_, Cl, V_*d*_, and C_2*min*_ are summarized in Table 3. All results were expressed as arithmetic mean ± standard deviation (SD). There was no significant difference in the pharmacokinetic parameters between group I and group II rats (*p* > 0.05), which were administered pemetrexed disodium. However, 20-day treatment of *Astragalus* extract decreased the AUC_(0−*t*)_, AUC_(0−*∞*)_, and MRT of gemcitabine (*p* < 0.05); meanwhile, the treatment with *Astragalus* extract increased the Cl of gemcitabine in rats (*p* < 0.05). The results might be caused by the metabolic characteristics of pemetrexed disodium and gemcitabine. The research indicated the elimination of pemetrexed disodium was primarily renal, with almost 80% of the dose recovered unchanged in the urine in the first 24 hours after administration [16]. However, the two important processes in the activation and elimination of gemcitabine were phosphorylation by deoxycytidine kinase and deamination by cytidine deaminase to the uracil metabolite [17]. According the above results, after 20-day treatment with the *Astragalus* extract, the activity of deoxycytidine kinase and cytidine deaminase would be changed in rats, which might lead to increase the clearance of gemcitabine.

In group I, rats were intravenously administered with 300 mg/kg pemetrexed disodium; in group II, rats were intravenously administered with 300 mg/kg pemetrexed disodium at day 21 of treatment with 3 g/kg/day *Astragalus* extract; in group III, rats were intravenously administered with 150 mg/kg gemcitabine; in group IV, rats were intravenously administered with 150 mg/kg gemcitabine at day 21 of treatment with 3 g/kg/day *Astragalus* extract. ^*∗*^*p* < 0.05 compared with group III.

## 4. Conclusion

In the present study, a simple, sensitive, and rapid HPLC method has been established and validated in rat plasma for simultaneous quantification of pemetrexed disodium and gemcitabine. Co-administration with *Astragalus* extract did not lead to an experimentally relevant effect on the pharmacokinetics of pemetrexed disodium. But the above results showed that *Astragalus* extract pretreatment could affect the metabolic characteristics of gemcitabine. Further investigations were needed to explicate the mechanism of drug-drug interactions and apply the findings in rats to human.

## Figures and Tables

**Table 1:** Precision and accuracy of pemetrexed disodium and gemcitabine determination in rat plasma (*n*=5).

Analytes	Spiked concentration (*μ*g/mL)	Intraday precision and accuracy		Interday precision and accuracy	
		Accuracy (%)	Precision (RSD, %)	Accuracy (%)	Precision (RSD, %)
Pemetrexed disodium	1	92.42	1.55	93.63	1.54
	200	109.29	2.00	105.72	4.34
	400	95.98	0.90	93.46	4.11

Gemcitabine	1	94.68	1.88	99.18	8.01
	200	98.61	1.41	94.93	3.44
	400	99.49	0.65	97.76	1.69

**Table 2:** Stability of pemetrexed disodium and gemcitabine during the storing and preparing procedures (mean ± SD, *n*=5).

Analytes	Spiked concentration (*μ*g/mL)	Measured concentration (*μ*g/mL)		
		6 h at room temperature	−20°C for 7 d	24 h at 4°C in storehouse
Pemetrexed disodium	1	0.97 ± 0.26	0.98 ± 0.09	0.97 ± 0.31
	400	352.86 ± 0.48	351.07 ± 0.43	354.73 ± 0.54

Gemcitabine	1	0.90 ± 0.01	0.99 ± 0.02	0.91 ± 0.01
	400	396.73 ± 1.65	392.75 ± 1.80	407.91 ± 2.77

**Table 3:** Pharmacokinetic (PK) parameters of pemetrexed disodium and gemcitabine in rats (mean ± SD, *n*=5).

PK parameters	Unit	Pemetrexed disodium		Gemcitabine	
		Group I	Group II	Group III	Group IV
AUC_(0−*t*)_	mg/L·min	24310.66 ± 6780.89	18083.12 ± 2679.02	15747.12 ± 497.11	12312.41 ± 594.21^*∗*^
AUC_(0−*∞*)_	mg/L·min	24368.58 ± 6849.85	18083.84 ± 2678.89	15976.18 ± 511.33	12489.59 ± 682.01^*∗*^
MRT	min	42.49 ± 18.02	36.20 ± 1.45	97.83 ± 5.82	84.37 ± 2.79^*∗*^
T_1/2*z*_	min	38.30 ± 7.64	25.03 ± 1.18	80.21 ± 1.22	80.00 ± 10.65
C_2*min*_	mg/L	621.28 ± 154.5	596.11 ± 117.83	171.96 ± 9.96	164.87 ± 8.28
V_*d*_	L/kg	0.36 ± 0.09	0.31 ± 0.05	2.18 ± 0.89	2.78 ± 0.34
Cl	L/min/kg	0.0067 ± 0.0018	0.0087 ± 0.0011	0.019 ± 0.0067	0.024 ± 0.0013^*∗*^

## Data Availability

The datasets generated during and analysed during the current study are not publicly available due to confidentiality agreement of the institution but are available from the corresponding author on reasonable request.
